# Event and Comment

**Published:** 1905-11-15

**Authors:** 


DENTAL REGISTER.

Vol. LIX. November 15, 1905. No. 11.

### EVENT AND COMMENT.

### The Cement Problem.

We have given considerable space to the publication of some experiments, designed to find scientific reasons for the failures of our cements in inlay work. The results are edifying if not conclusive and point the way to further researches, which shall give the data necessary for the further improvement of this form of filling material. We may never find a satisfactory cement filling material, but there is no doubt but that we shall make great improvement on the present product. It seems to us that the cements we now have should produce more satisfactory results under more scientific handling, and that this feature of the investigation should not.be passed over as it has an important bearing. Every dentist meets cement filling that have stood hard usage for many years with but slight disintegration, while the large majority of cement fillings in exposed situations do not last a year. We are in the habit of attributing this to the cement or conditions of the oral secretions. Strongly alkaline oral secretions may dissolve away the best cements or fermenting products may also disintegrate them, but from comparing results from the various methods of manipulating cements, we believe that the average dentist does not mix his cement properly. We have seen dentists mix cement on a rough porcelain slab, not over two inches square, with a narrow spatula that was so flexible that no thorough spatulation could be made. We have also seen it mixed on a slab that was smeared with many previous mixes, which greatly retarded thorough mixing. Then again such mixes are carried to the cavities with instruments that are not at all adapted for quick and thorough placing. With such operators any form or condition of cavity is good enough for amalgam or cement. We have recently found in the dental supply houses a glass mixing slab that ought to be in every dental office. It is three inches wide, six inches long and one inch thick, and is nicely ground and polished on all sides, making it the ideal mixing slab. It is perfectly smooth and easily kept clean, and will maintain a uniform temperature. If a large bladed spatula is used for mixing the cement on such a slab, and plenty of time and strength be intelligently employed, we believe that better results would come from the use of the cements that are now available.

**New Features in Dental Legislation.**

The State of Nebraska has just enacted a new law for the control of the practice of dentistry, which contains new features that express the trend of thought as to dental qualifications at the present time. One of the important features of this law is the placing of the examiners under the control of the State Board of Health, which appoints the Board of Examiners and assumes the responsibility of enforcing the law. There are to be five examiners appointed for terms of five years, and each candidate must be endorsed by fifty legalized practitioners of dentistry before the Board of Health can consider him. Both of these features are good and ought to help in securing a good board of examiners and the enforcement of the laws. The law defines qualifications for examination for registration on the basis of graduation from a reputable dental college, one which conforms to the standards set by the National Board of Examiners, or on the basis of five years of legalized practice in another state. Any one who is a graduate of a high school can secure a certificate from the board before endenturing himself as an office student for a term of five years, and will, at the expiration of his endenture, be permitted to take the examination for license to practice. Dental Colleges located in the State may register their graduates without examination, provided they permit the State Board of Examiners to conduct or be present at the final examinations of their graduates. This is a very wise provision and if properly carried out, would practically put the colleges under the supervision of the State Board. Another important feature which is not new exactly, is that the license of any man who is guilty of gross immoral conduct, can be revoked by the State Board of Health. The California and Illinois laws have this feature, which has never been tested out legally and which may not be constitutional.

**The First Molar.**

In a lecture recently given by a prominent orthodontist, he characterized the extraction of the first molar teeth for the correction of irregularities as "*the Crime of Dentistry.*" He made this statement in discussing the method advocated by many that the extraction of these teeth would give more space for the subsequently developing teeth, if the first molars were extracted not later than the age of eleven years. He illustrated by lantern slides the results claimed by dentists who had written essays on this subject, reproducing pictures of their cases. It was very evident from the pictures shown that there was not a single case of even good occlusion of the teeth, and the pictures shown revealed the fact that the teeth were not only in mal-occlusion, but that they had so shifted that the gum festoons were left exposed to the food impact during the function of mastication, resulting in irritated or hypertrophied gums. He went further also to show that the loss of any tooth was almost a crime. He advocated the treatment and preservation of the first molars, especially until the full denture had erupted. The illustrations shown were certainly convincing and we should think his auditors would have a nervous chill after extracting a first molar tooth that could be saved by any possible means. There is no question but we neglect our little patients too much. If the deciduous teeth were carefully inspected and protected from caries at the proper time, there would be fewer cases of mal-occlusion. It is high time dentists were taking more pains to impress upon parents the value of early attention to the deciduous teeth. If the deciduous teeth could be kept until the corresponding permanent teeth were ready to take their places, and all first molars were saved until twenty-one years of age, there would be fewer cases of mal-occlusion. How long will it take dentists to learn that every tooth, yea, every cusp of every tooth is essential to the best welfare of his patients.

**The Army Dentist,**

We print in this issue an abstract from the annual report of a contract dental surgeon in the U. S. Army, which, we think, interesting and instructive as it gives the detail itinerary of the army dentist and the need for him in the army posts of our own country, where the soldiers are not in active service. And yet we find that this army dentist has been kept very busy rendering important services for the comfort and welfare of the soldiers and officers. This dentist treated over eight hundred persons, and performed an average of three operations for each person. When we consider that this would constitute a good practice for a private practitioner, and deduct the time the army dentist must have lost in traveling from one station to another, it will be apparent to everyone that he must have been kept busy. This is also further shown by the fact that he averaged nine sittings each day he worked. The work ranged all the way from curing toothache and tooth filling to the treatment of abscesses and necrotic conditions. This report may also be of interest to those who contemplate entering this service. It certainly justifies the employment of army dentists, and ought to have great weight in inducing the authorities to put this work on a more substantial basis. There is a bill before Congress now to put dentists in the army and navy on the commissioned basis, in which every member of the professios ought to feel obligated to do what he can to enlist the interest of his Congressmen. If the work could be put on such a basis it would not only secure a better class of men for the service, as they would have a dignified rank, but it would naturally secure greater proficiency. It is not probable that every post or every warship can have its dentist, although each one should have enough such officers and should be appointed to give the soldiers the best service that is practicable. The soldier enlists for a term of years and the service should be all that the period demands, while the officers should have more careful service, as they enlist for life.

**Qualification for Practicing Dentistry in England.**

The English dental registration laws exclude all dentists educated in foreign countries from legally practicing in the British Empire; but allows the profession to be degraded by permitting commercial institutions which are very common everywhere, under the direction of irresponsible and uneducated people. The following taken from *The Dental Surgeon*, is a sample of a new corporation:

"REGISTERED May 30. Mrs. Lina Clare, Ltd. Capital, £2,000, in £1 shares. Object, to carry on hygienic complexion treatment, electrolysis, manicure, chiropody, massage, treatment of the scalp, etc., hairdressing, manufacturing and dealing in perfumes and toilet requisites and the like, *and to act as dentists*, chemists, druggists, photographers, etc. No initial public issue. The first directors (to number not less than two nor more than five) are to be appointed by the signatories. Qualification, £50. Remuneration as fixed by the company. Registered office: 50 Maddox Street, Regent Street, W."

